# The need for environmental surveillance to understand the ecology, epidemiology and impact of *Cryptococcus* infection in Africa

**DOI:** 10.1093/femsec/fiab093

**Published:** 2021-07-01

**Authors:** Hannah M Edwards, Massimo Cogliati, Geoffrey Kwenda, Matthew C Fisher

**Affiliations:** MRC Centre for Global Infectious Disease Analysis, Imperial College School of Public Health, Imperial College London, Norfolk Place, London W2 1PG, UK; Dip. Scienze Biomediche per la Salute, Università degli Studi di Milano, Via Pascal 36, 20133 Milano, Italy; Department of Biomedical Sciences, School of Health Sciences, University of Zambia, Ridgeway Campus, PO Box 50110, Lusaka, Zambia; MRC Centre for Global Infectious Disease Analysis, Imperial College School of Public Health, Imperial College London, Norfolk Place, London W2 1PG, UK

**Keywords:** fungi, genomics, ecology, epidemiology, microbiology, evolutionary biology

## Abstract

Our understanding of the pathogenic yeasts *Cryptococcus neoformans* and *Cryptococcus gattii* has been greatly enhanced by use of genome sequencing technologies. Found ubiquitously as saprotrophs in the environment, inhalation of infectious spores from these pathogens can lead to the disease cryptococcosis. Individuals with compromised immune systems are at particular risk, most notably those living with HIV/AIDS. Genome sequencing in combination with laboratory and clinical studies has revealed diverse lineages with important differences in their observed frequency, virulence and clinical outcomes. However, to date, genomic analyses have focused primarily on clinical isolates that represent only a subset of the diversity in the environment. Enhanced genomic surveillance of these yeasts in their native environments is needed in order to understand their ecology, biology and evolution and how these influence the epidemiology and pathophysiology of clinical disease. This is particularly relevant on the African continent from where global cryptococcal diversity may have originated, yet where environmental sampling and sequencing has been sparse despite harbouring the largest population at risk from cryptococcosis. Here, we review what scientifically and clinically relevant insights have been provided by analysis of environmental *Cryptococcus* isolates to date and argue that with further sampling, particularly in Africa, many more important discoveries await.

## Teaser

We summarise important insights unveiled by environmental sampling and genomics of the pathogenic fungi, Cryptococcus, but argue there is still much to learn with increased focus in this area.

## INTRODUCTION

Fungal diseases cause a considerable and underappreciated burden of disease worldwide (Bongomin *et al*. 2017). A vast diversity of fungi exist as saprotrophs in the environment, some of which can cause opportunistic disease in at-risk humans—these fungi are often known as ‘sapronoses’. With current climate trends, fungal sapronoses are expected to present an increasing risk and burden to human health since they often thrive in warm and wet conditions (Garcia-Solache and Casadevall 2010). Fungal sapronoses are largely made up of species of moulds in the phylum, Ascomycota, however, the sister phylum of Basidiomycota includes the species complexes of *Cryptococcus neoformans* and *Cryptococcus gattii*. Unlike the majority of the Basidiomycota which are filamentous, *Cryptococcus* spp. are yeasts which are cosmopolitan in environments worldwide and can cause the disease cryptococcosis when aerosolised spores and/or desiccated yeast cells are inhaled by a susceptible individual (Velagapudi *et al*. 2009; Walsh *et al*. 2019). The resulting infection can affect any organ but often manifests as an acute pneumonia or a highly fatal meningitis (Kronstad *et al*. 2011). These fungi predominantly affect immunocompromised individuals, particularly those with HIV/AIDS among whom they are estimated to cause 223 100 new cases and more than 181 000 deaths globally per year, three quarters of which (162 500 new cases and 135 900 deaths) are in sub-Saharan Africa (Rajasingham *et al*. 2017). Other estimates place the incidence of cryptococcal meningitis among the general population in Africa at 4.8 per 100 000 during the years 1990 to 2017 (Nyazika *et al*. 2019). In comparison, Asia and Pacific region has the second-highest burden of disease with an estimated 43 200 new cases and 39 700 deaths annually, while Europe has just 4400 and 1800 new cases and deaths, respectively, per year (Rajasingham *et al*. 2017).

As single-celled yeasts, the increasing availability and affordability of genome sequencing has broadened our understanding of these pathogens and, in combination with complementary laboratory and clinical studies, has revealed a genetically diverse set of lineages. Within *C. neoformans* there are five main molecular types: VNI, VNII and VNB, collectively known as *C. neoformans var. grubii*; VNIV, also known as *C. neoformans var. neoformans*; and VNIII, a hybrid of the two varieties. Within *C. gattii* there are the lineages VGI, VGII, VGIII, VGIV and the more recently discovered and described, VGV (Farrer *et al*. 2019). Few and rare inter-species hybrids have also been reported (Bovers *et al*. 2006, 2008; Aminnejad *et al*. 2012). The diversity between these lineages revealed by whole-genome sequencing (including single nucleotide polymorphisms (SNPs), insertions and deletions (INDELs) and genomic rearrangements (Desjardins *et al*. 2017; Rhodes *et al*. 2017b; Vanhove *et al*. 2017)) has led to proposals to elevate these molecular types to species level (Hagen *et al*. 2015).

Since these fungi are acquired from nature, environmental sampling and genomic analysis is key to understanding their diversity, biology, ecology and epidemiology. To date, such analysis of environmental *Cryptococcus* spp. has, however, been limited, particularly in Africa (Cogliati 2013). Yet, it is this region that warrants greater investigation; not only does southern Africa hold the largest population that are at-risk from cryptococcosis due to the high number of HIV/AIDS-infected individuals (Perfect and Bicanic 2015; Oladele *et al*. 2017; Rajasingham *et al*. 2017), it is also hypothesised as being home to the ancestral diversity from which more globalised lineages evolved (Litvintseva *et al*. 2011). While much has already been learned from the limited number of environmental isolates gathered here, we argue that far more can be discovered by focusing more attention on environmental cryptococcal genomics across this region.

## GLOBAL SAMPLING AND SEQUENCING EFFORT TO DATE

**Figure 1.**
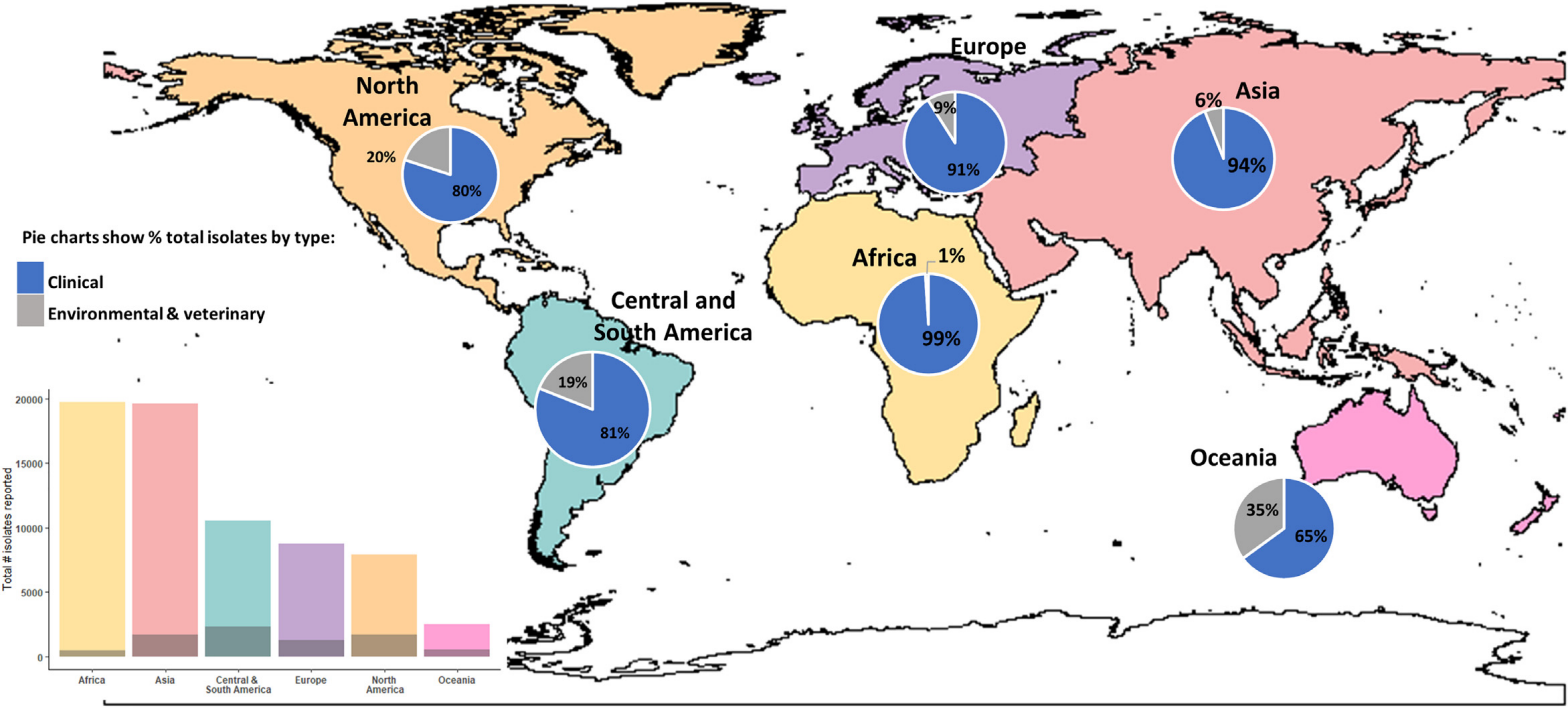
Reported isolations of *C. neoformans* and *C. gattii* across each continental region up to Cogliati's 2013 review. Pie charts show distribution of clinical and environmental/veterinary sources of isolation. Bar chart shows total number of isolates reported with shaded regions as the number that were examined for molecular type. Despite data being from 2013, general patterns and proportions remain true.

**Figure 2.**
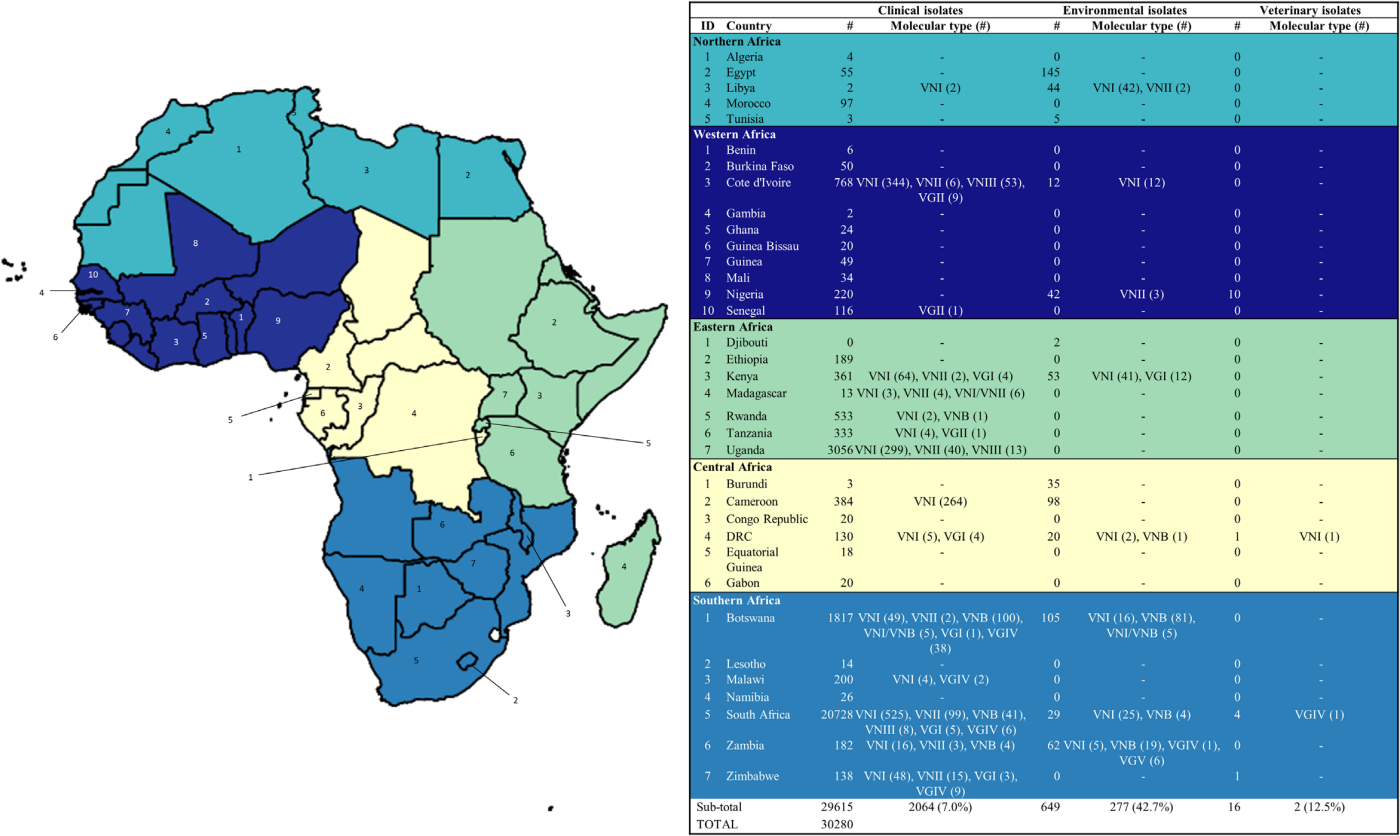
Clinical, environmental and veterinary isolations (published) of *C. neoformans* and *C. gattii* by country and region across Africa.

Our understanding of the distribution of cryptococcal genotypes is directly influenced both by the amount of clinical and environmental sampling conducted and by the proportion of sampled isolates that have been molecular typed. Cogliati's 2013 review found there were 69 022 isolates of *C. neoformans* and *C. gattii* reported globally, with the vast majority collected from Africa and Asia, and fewest from Oceania (Fig. 1) (Cogliati 2013). Of these isolates, less than 10% were from environmental or veterinary sources (as opposed to clinical), and less than 12% had been examined for molecular type. Of all regions, Africa had the highest volume of clinical isolates collected, as may be expected given this area carries the greatest burden of clinical disease. However, this region also has the lowest proportion of isolates that have been molecular typed. For our current review we updated Cogliati's 2013 study for Africa and found a total of 30 280 isolates reported of which 8% (n = 2343) were examined for molecular type and only 2% (n = 649) were collected from the environment (Fig. 2, Table 1 and Supplementary Table). The proportion of environmental isolates that are molecular-typed is higher at 43% because these are predominantly from research studies, as opposed to most clinical isolates, which are collected in routine diagnoses.

**Table 1.** Environmental *Cryptococcus neoformans* and *Cryptococcus gattii* isolations from countries in Africa.

Country (total # isolates)	Region	Environmental source	# isolates recovered	Species, variety, serotype, molecular type (as reported)	Reference
Libya (44)	Tripoli	Pigeon droppings	32	*C. neoformans* var. *grubii*, A, VNI	Ellabib *et al*. 2016
			1	*C. neoformans* var. *grubii*, A, VNI	
		*E. camaldulensis*	2	*C. neoformans* var. *grubii*, A, VNII	
		*Olea europaea*	9	*C. neoformans* var. *grubii*, A, VNI	
Tunisia (5)	Sfax region	*E. camaldulensis*	1	*C. neoformans* species complex	Mseddi *et al*. 2011
		*E. camaldulensis*	2	*C. gattii* species complex	
		Almond tree (*Prunus dulcis*)	2	*C. gattii* species complex	
Egypt (145)	Tanta	*E. camaldulensis*	1	*C. gattii* species complex	Mahmoud 1999
	Qutur	*E. camaldulensis*	2	*C. gattii* species complex	
	Gharbia Governatorate	Avian droppings	95	*C. neoformans* species complex	
	Nile delta	Pigeon droppings	30	*C. neoformans/C. gattii*	Refai *et al*. 1983
	Giza	*E. camaldulensis*	3	*C. neoformans* var. *grubii*	Elhariri *et al*. 2016
	Cairo	*E. camaldulensis*	2	*C. neoformans* var. *grubii*	
	Al-Sharqia	*E. camaldulensis*	5	*C. neoformans* var. *grubii*	
	Elmenofia	*E. camaldulensis*	3	*C. neoformans* var. *grubii*	
	Abulnomorous	Ground water	3	*C. neoformans* var. *grubii*	Elfadaly *et al*. 2018
	Shabramant	Ground water	1	*C. neoformans* var. *grubii*	
Kenya (53)	Nairobi	Avian droppings	23	*C. neoformans* var. *grubii*, VNI	Kangogo *et al*. 2015
			5	*C. gattii*, VGI	
		Tree swabs	5	*C. neoformans* var. *grubii*, VNI	
			7	*C. gattii*, VGI	
		Chicken cages	5	*C. neoformans* var. *grubii*, VNI	
		Garbage dumping	6	*C. neoformans* var. *grubii*, VNI	
		Soil	2	*C. neoformans* var. *grubii*, VNI	
Djibouti (1)	Djibouti	Pigeon droppings	2	*C. neoformans/C. gattii*	Pal 2015
Cameroon (98)	West region	Pigeon droppings and bat guano	57	*C. gattii* species complex	Dongmo *et al*. 2016
			41	*C. neoformans* species complex	
Ivory Coast (12)	Adjamé	Pigeon droppings	12	*C. neoformans* var. *grubii*, A, VNI	Kassi *et al*. 2018
Nigeria (41)	Southeastern Nigeria	Pigeon droppings	39	*C. neoformans/C. gattii C. neoformans species complex C. neoformans* var. *grubii*, VNII	Nweze *et al*. 2015
	Jos	Pigeon droppings	3		
					Nnadi *et al*. 2016
Democratic Republic of Congo (20)	Zaire	House dust	1	*C. neoformans* var. *grubii*, A, VNI	Boekhout *et al*. 2001
		Wood	1	*C. neoformans* var. *grubii*, A, VNI	
		Wood	1	*C. neoformans* var. *grubii*, A, VNB	
	Kinshasa	House dust	2	*C. neoformans* var. *grubii*, A	Varma *et al*. 1995
	Kinshasa	House dust	4	*C. neoformans* species complex	Swinne *et al*. 1986
		House air	2	*C. neoformans* species complex	
		Chicken droppings	2	*C. neoformans* species complex	
		Pigeon droppings	7	*C. neoformans* species complex	
Burundi (35)	Bujumbura	Environment	15	*C. neoformans* species complex	Varma *et al*. 1995
	Bujumbura	Patient's house	7	*C. neoformans* species complex	Swinne *et al*. 1989
	Bujumbura	House dust	13	*C. neoformans* species complex	Swinne *et al*. 1991
Zambia (32)	Zambesi and Miombo woodlands	Trees	5	*C. neoformans* var. *grubii*, VNI	Vanhove *et al*. 2017
			19	*C. neoformans* var. *grubii*, VNB	
			31	*C. gattii* species complex	
	Miombo woodlands	Hyrax midden	4	*C. gattii*, VGV	Farrer *et al*. 2019
			1	*C. gattii*, VGIV	
		Tree hole	2	*C. gattii*, VGV	
Botswana (105)	Gaborone	Pigeon droppings	3	*C. neoformans* var. *grubii*, A, VNI	Litvintseva *et al*. 2011
	Gaborone	Tree bark	2	*C. neoformans* var. *grubii*, A, VNI	
	Tuli block	Mopane tree	15	*C. neoformans* var. *grubii*, A, VNB	
	Tuki block	Mopane tree	4	*C. neoformans* var. *grubii*, A, VNI	
	Tuli block	Soil	2	*C. neoformans* var. *grubii*, A, VNI	
	Tuli block	Soil	1	*C. gattii*, B	
	Tuli block	Baobab	2	*C. neoformans* var. *grubii*, A, VNB	
	Francistown, Gaborone, and Maun	Trees and bird excreta	5	*C. neoformans* var*. grubii*, VNI	Chen *et al*. 2015
			64	*C. neoformans* var*. grubii*, VNB	
			5	*C. neoformans* v*. grubii*, VNI/VNB	
			2	*C. gattii* species complex	
South Africa (29)	Durban	Pigeon droppings	20	*C. neoformans* var. *grubii*, A, VNI	Litvintseva *et al*. 2011
	Johannesburg	Soil	2	*C. neoformans* var. *grubii*, A, VNI	
	Parys	Pigeon droppings	3	*C. neoformans* var. *grubii*, A, VNI	
	Zeerust	Eucalyptus tree	2	*C. neoformans* var. *grubii*, A, VNB	
	Zeerust	Soil	2	*C. neoformans* var. *grubii*, A, VNB	

## GLOBAL DISTRIBUTION OF GENOTYPES AND AFRICA IN CONTEXT

**Figure 3.**
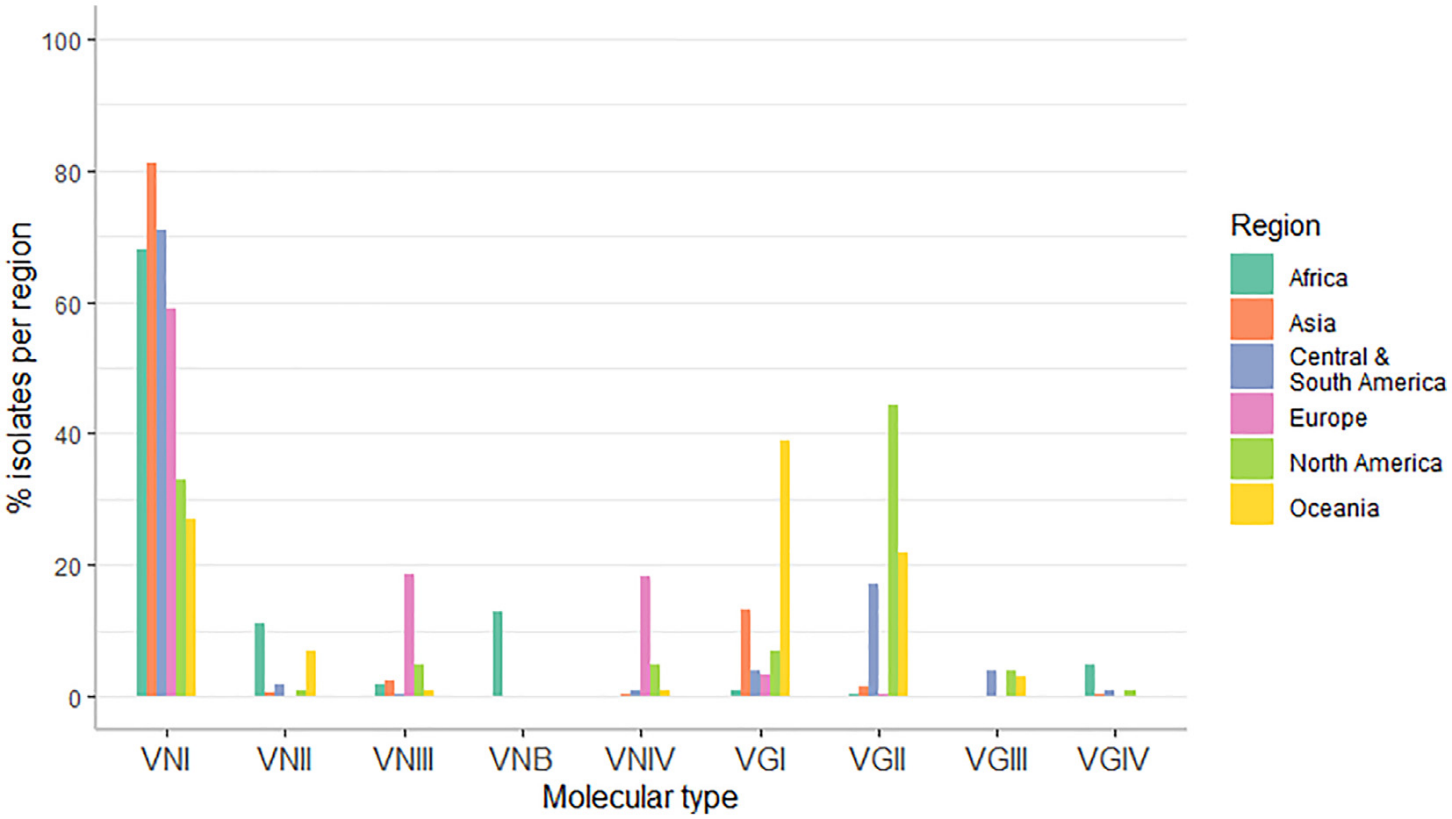
Distribution of the main *C. neoformans* and *C. gattii* molecular types identified over different global regions, as reported in Cogliati 2013. Since this review additional molecular types have been identified, including VNB in Central and South America and VGV in Africa, however general distribution patterns remain true.

Figure 3 summarises the distribution of molecular types by global region based on Cogliati's 2013 review (Cogliati 2013). Since this review, isolates of the VNB molecular type have also been identified from six clinical cases and one environmental sample in South America and the new *C. gattii* lineage, VGV, was identified from environmental sources in Africa (Rhodes *et al*. 2017b; Farrer *et al*. 2019). However, the overarching patterns within the data documented by Cogliati remain.

The VNI molecular type has been isolated from all regions and is the dominant molecular type across all except North America and Oceania where VGI and VGII dominate, respectively (Fig. 3). The majority of global clinical disease is caused by infection with *C. neoformans* var. *grubii* which makes up approximately 95% of all cryptococcosis cases worldwide (Maziarz and Perfect 2016). Of these, the vast majority are due to infection with the VNI molecular type. VNI is frequently isolated from the environment where it is associated with trees, pigeon and other bird guano, and with urban sites including churches and dwellings (Table 2) (Litvintseva *et al*. 2011; Chen *et al*. 2015; Kangogo *et al*. 2015; Nweze *et al*. 2015; Ellabib *et al*. 2016; Nnadi *et al*. 2016; Kassi *et al*. 2018). VNB, on the other hand, has only been isolated from Southern Africa and, more recently, South America, from a small number of clinical isolates and from arboreal tree species.

**Table 2.** Sources of environmental and veterinary isolates of *C. neoformans*and *C. gattii* in each global region.

	Sources of environmental and veterinary isolates*	
Region	*C. neoformans*	*C. gattii*
Oceania	Environmental: *Eucalyptus camaldulensis*, pine needles	Environmental: *Eucalyptus camaldulensis*, *Eucalyptus tereticornis*, *Syncarpia glomulifera*, insect frass, olive seedlings, plant debris
	Veterinary: cat, dog, horse, koala, ferret, *Potorous gilbertii*	Veterinary: kiwi, cat, dog, horse, sheep, cow, koala, quokka, cockatoo, ferret, *Potorous tridactylus*, echidna, African grey parrot, dolphin
Asia	Environmental: Mostly from pigeon and other bird excreta, less frequently from trees including *Eucalyptus*, *Tamarindus arjuna*, *Tamarindus indica*, *Cassia fistola*, *Syzygium cumini*, and *Ficus religiosa;* and some fruit and vegetables (tomato, carrot, banana, eggplant, papaya, apple, guava)	Environmental: Trees including *Syzgium cumini*, *Mimusops elengi*, *Azadirachta indica*, *Acacia nilotica*, *Cassia fistola*, *Manikara hexandra*, *Polyalthia longifolia*, *Eucalyptus camaldulensis*, *Tamarindus indica*, *Cassia marginata*, and *Mangifera indica*
	Veterinary: cat, dog, bandicoot	Veterinary: koala
Africa	Environmental: Pigeon and bird excreta, soil, house dust, trees including Eucalyptus camaldulensis, mopane, baobab	Environmental: Soil, Eucalyptus camaldulensis, almond tree
	Veterinary: N/A	Veterinary: cheetah
Europe	Environmental: Mostly from pigeon, bird and bat guano, and red fox faeces. Few from trees including *Eucalyptus camaldulensis* and oak tree	Environmental: mostly from trees including *Eucalyptus camaldulensis*, Douglas tree, carob tree, stone pine
	Veterinary: cat, dog, magpie, striped grass mouse, degu.	Veterinary: ferret, goat
Central and South America	Environmental: pigeon and bird excreta, soil, dust, contaminated dwellings, Eucalyptus tree, almond tree, kassod tree, pink shower tree, *Caesalpinia peltophoroides*, *Anadenanthera peregrine*	Environmental: soil, dust, *psittaciformes* bird excreta, *Eucalyptus camaldulensis*, almond tree, kassod tree, pottery tree, jungle tree, *Corymbia ficifolia*, *Cephalocereus royenii*
	Veterinary: insects, bull, sheep	Veterinary: cheetah, goat, psittacine birds
North America	Environmental: Mainly from pigeon droppings, some from fruit and vegetables	Environmental: Soil, trees, air, water
	Veterinary: ferret	Veterinary: dog, cat, horse, ferret, birds, alpaca, parrots

*information taken from Cogliati^2013^

*Cryptococcus gattii* rarely causes clinical disease and was previously thought to be restricted to tropical and sub-tropical regions, where it is associated predominantly with arboreal tree species including Eucalyptus, olive trees, and dry-tropical miombo (*Brachystegia* sp.) (Ellis and Pfeiffer 1990; Pfeiffer and Ellis 1992; Mseddi *et al*. 2011; Cogliati *et al*. 2016; Vanhove *et al*. 2017) (Table 2). However, the molecular type, VGII, has been the cause of recent outbreaks in more temperate and developed areas of the world, such as in PNW, Vancouver and Oregon (Byrnes and Marr 2011) and has become the dominant molecular type reported here owing to intensive clinical and environmental surveillance in regions affected by the outbreak (Bartlett, Kidd and Kronstad 2008; Billmyre *et al*. 2014; Engelthaler *et al*. 2014). VGII has been isolated across the Pacific North West region from trees, sea water and marine animals.

In Africa, clinical infection is most commonly associated with VNI infection, although a high diversity of lineages have been identified from clinical cases, especially in Southern Africa (Fig. 2 and Supplementary Table). This region has also uncovered high diversity from the environment, with VNB being most commonly isolated, followed by VNI and several lineages of C. gattii (Fig. 2 and Table 1). Despite the limited sequencing, the diversity of molecular types in southern Africa is one of the factors supporting the ‘out-of-Africa’ hypothesis which postulates that *Cryptococcus* diversified in Africa prior to subsequent global spread. However, sampling and molecular-typing have been even more limited in regions other than Southern Africa, including Central Africa which shares a large border with the southern region and thus shares some of the ecological habitats which favour cryptococcal growth and harbour diversity (Fig. 2).

## WHAT HAVE ENVIRONMENTAL ISOLATES TAUGHT US AND WHAT COULD THEY YET STILL REVEAL?

Despite the limited volume of environmental sampling and genomics analysis conducted to date, the genomes and associated biology of *Cryptococcus* spp. recovered from environmental sources has provided useful insights into various aspects of *Cryptococcus* evolution, virulence and epidemiology. The focus on clinical cases is understandable but, we argue, that combining clinical analysis with increased focus on what exists in the environment can help answer some of the key knowledge gaps in understanding the impact of this opportunistic infection. We group these insights and remaining areas of research under four key themes, described here in turn: (i) evolutionary origins, speciation and spread of genotypes, (ii) biology of virulence, (iii) exposure risk and epidemiology and (iv) emergence of drug resistance. We finally discuss some of the challenges in environmental sampling and modelling of *Cryptococcus*. Although we discuss global research, we highlight where we believe environmental sampling can answer knowledge gaps particularly pertaining to the African context where the highest burden of disease is concentrated and thus the biggest gains are to be made.

## EVOLUTIONARY ORIGINS, SPECIATION AND SPREAD OF GENOTYPES

Since the genotypes of *C. neoformans* and *C. gattii* that cause clinical infection are a subset of what occurs in the environment, environmental sampling will reveal the true extent of the taxonomic diversity within each species complex. The significance of this was recently demonstrated with the discovery of an entirely new lineage of the *C. gattii* species complex, VGV, from environmental sampling conducted in Zambia in 2013 (Farrer *et al*. 2019). This discovery demonstrates there may yet be more diversity to discover given greater surveillance effort in new and more varied ecotypes and ecoregions. Understanding the full taxonomic diversity of *Cryptococcus* is not only of general biological interest; subsequent phylogenetic and population genomics analyses provide important insights into evolutionary origins, speciation and genotype flow. For example, phylogenetic analyses of loci from environmental genomes in South Africa and Botswana showed a high proportion of *C. neoformans* isolates from African arboreal trees belong to the genetically diverse and sexual lineage, VNB, which is ancestral to the globalised and asexual VNI and VNII lineages. This finding has been used to propose an ‘out-of-Africa’ hypothesis to account for the current distribution of *C. neoformans* genotypes (Litvintseva *et al*. 2011). Conversely, evidence to date suggests that lineages of the *C. gattii* species complex appear to originate from South America and that the species complexes themselves may have diverged 80–100 million years ago at the time of the breakup of the Pangean supercontinent (Hagen *et al*. 2013; Casadevall *et al*. 2017). Although geography can help explain patterns of speciation, closely related genotypes of both *C. neoformans* and *C. gattii* have also been found on separate continents, suggesting that relatively recent long-distance dispersal events occur (Ashton *et al*. 2019). Specifically, the highly virulent VGII lineage is hypothesised to have spread to the North American Pacific Northwest (PNW) 70–90 years ago from Brazil, possibly vectored by trade along shipping routes and assisted by passive dispersal in ocean currents (Engelthaler and Casadevall 2019).

Population genetics comparing environmental and clinical isolates is a powerful approach in not only understanding these long-distance dispersal events, but also the rate at which genotypes move across smaller scales. In Europe, the geographical distribution of clinical and environmental isolates together with analysis of spatial patterns of gene-flow allowed inference of how the main VNI sequence types circulate and highlighted Germany and Italy as the ‘fulcrum’ of diffusion of both endemic and imported genotypes (Cogliati *et al*. 2019). At finer-scales, genome-sequencing and phylogenetic analysis is now being used to investigate sources of exposure leading to cryptococcosis, for instance in recent attempts to link hospital environments to nosocomial outbreaks of the disease (Farrer *et al*.^2021^). Similar increased sampling and sequencing of environmental isolates in Africa would describe the spatial genetic structure of lineages and genotypes throughout the continent. These data would, at last, provide a baseline from which a more nuanced understanding of the epidemiology of exposure and infection for the large at-risk population of people living with HIV/AIDS in Africa could be developed.

## BIOLOGY AND EMERGENCE OF VIRULENCE

Comparing the biology of both clinical and environmental cryptococcal isolates lends insight into differences in virulence between isolates and genotypes as well as what genomic mechanisms can generate diversity that may explain the emergence of virulent phenotypes. The ability of *Cryptococcus* to adapt to selective pressures in the environment is linked to plasticity of its genome which allows changes in ploidy, microevolution and hypermutator states leading to phenotypic switching (Guerrero *et al*. 2006; Jain and Fries 2008; Magditch *et al*. 2012; Rhodes *et al*. 2017a), as well as its ability to recombine it's genome through recombination. The yeast is able to mate both bisexually between two cells of opposing mating types (MAT-a and MAT-α) as well as unisexually between two members of the same mating type, with unisexual reproduction still leading to diverse progeny and biologically important since MAT-a cell types are rare (Nielsen *et al*. 2003; Ni *et al*. 2013; Phadke *et al*. 2014; Fu *et al*. 2015; Sun *et al*. 2019). Such genomic mechanisms may also contribute to the emergence and spread of global virulent phenotypes. For example, evidence has implicated both microevolution (via a transient mutator phenotype) and sexual reproduction (either unisexual or bisexual) in the emergence of the virulent VGII strains responsible for the PNW outbreak (Billmyre *et al*. 2014). Although primarily a haploid organism, changes in ploidy and cell size increases, such as seen in polyploid titan cells, can occur in response to environmental stressors and during human infection this can result in enhanced virulence, dissemination and survival within the host (Gerstein *et al*. 2015; Hommel *et al*. 2018; Zhou and Ballou 2018).

Humans are dead-end hosts for *C. neoformans* and *C. gattii*; pathogenesis is thus considered to be an ‘accidental’ by-product of traits that have evolved in response to natural selection in the environment rather than selection for virulence within a mammalian host (Casadevall 2008; May *et al*. 2016). These attributes thus have a ‘dual-use’ survival value that is manifested both in the environment as well as the accidental host (Casadevall, Steenbergen and Nosanchuk 2003). For example, a complex thick-walled polysaccharide capsule protects against desiccation and predation by amoebae in the environment as well as phagocytosis by macrophages in the host; melanin production protects against ultraviolet light and temperature fluctuations in the environment as well as resistance to oxidative stress, body temperature, the immune system and drug treatment pressures in the host; laccase production aids lignin degradation in the environment as well as protecting against oxidative bursts in the host (Williamson 1997; Guerrero *et al*. 2006; Perfect 2006; Jain and Fries 2008; Magditch *et al*. 2012; Rhodes *et al*. 2017a; Casadevall *et al*. 2019; Zaragoza 2019). The capacity for virulence that is independent of the requirement for animal hosts to aid survival and replication has been termed ‘ready-made’ virulence, as opposed to virulence that is selected for through dependence and/or symbiosis with the host (Casadevall, Steenbergen and Nosanchuk 2003). This hypothesis does not explain the whole story, however, since most species of *Cryptococcus* (and other environmental fungi) do not appear to infect mammalian hosts yet likely experience similar environmental pressures as *C. neoformans*/*C. gattii* (Casadevall, Steenbergen and Nosanchuk 2003).

Although it is accepted that virulent genotypes are acquired from the environment and that virulence factors are largely a result of adaptations to environmental pressures, few studies have specifically compared the virulence of environmental isolates to that of clinical isolates. Since some molecular types are found more frequently in clinical cases than the environment, and *vice versa*, there must either be biological differences in virulence between molecular types, or differential exposure of the susceptible human population to each molecular type. For example, the division of VNB into two distinct phylogenetic clades, VNBI and VNBII, characterised notable phenotypic differences between these two groups. In Botswana, VNBII was enriched for clinical isolates relative to VNBI which contained a far higher number of environmental isolates (Desjardins *et al*. 2017). The same trend was seen by a separate study in Zambia where VNBII (which the authors denoted VNB-A) comprised a mix of environmental and clinical isolates while VNBI (denoted VNB-B) was entirely environmental in origin (Vanhove *et al*. 2017). The comparison is more complex since, although evidence is limited, differences in virulence can occur not only between lineage types but also between environmental and clinical isolates of the same lineage type. Perhaps surprisingly, high-throughput phenotyping showed that VNBI environmental isolates were more resistant to oxidative stress and more heavily melanized that VNBI clinical isolates. Here, lack of melanisation was associated with loss-of-function mutations in the *BZP4* transcription factor and likely reflects a greater breadth of selective pressures in the environment than in the human host (Desjardins *et al*. 2017). This may suggest, then, that the lower incidence of VNBI clinical cases is due to more limited exposure to their infectious propagules rather than a lack of intrinsic ability to infect the human host. However, earlier studies found differential ability of environmental strains of *C. neoformans* to cause disease in murine models (Da Silva *et al*. 2006) and lower virulence than clinical isolates (Fromtling, Abruzzo and Ruiz 1989), although these studies did not distinguish molecular type. Litvintseva & Mitchell (Litvintseva and Mitchell 2009) found that only one VNI isolate of 11 environmental isolates of *C. neoformans* (including 10 VNI and 1 VNII) caused infection in mice up to 60 days post-infection, whereas 7 of 10 clinical isolates were lethal at median times of 19 and 40 days (lethal clinical isolates included 6/7 VNI and 2/3 VNII).

These intriguing findings suggest that genetically encoded mechanisms driving emergence of virulent phenotypes may be complex and it is yet to be conclusively determined what genetic and/or epigenetic factors may play a role. If virulence is a result of adaptation to the yeast's local environment then it may be determined by the micro-ecological niche that each isolate occupies, resulting in differences between apparently similar populations. Further dissection of the eco-evolutionary basis of cryptococcal virulence is certainly warranted and may provide insight into how to better manage infection when it does occur.

## HOW ECOLOGY CAN SHAPE CLINICAL EPIDEMIOLOGY AND EXPOSURE

Environmental genomic surveillance also helps explain patterns of clinical disease and risk of human exposure to *Cryptococcus*. It is hypothesised that growth on bird guano as a key niche may have led to VNI's widening global distribution in concert with bird domestication and association with urban locales (Nielsen, De Obaldia and Heitman 2007). In comparison, VNB environmental isolates have only been isolated from arboreal trees in rural Africa and, once, from Brazil. In turn, VNB infections are rare and restricted to these areas of Africa and South America, suggesting that patients are acquiring VNB infections as a consequence of their exposures to these arboreal reservoirs (Litvintseva *et al*. 2011; Vanhove *et al*. 2017; Rhodes *et al*. 2017b). The exact extent and type of VNB arboreal reservoir in South America remains unknown, however.

How ecological niche has shaped *C. gattii* distribution is less clear since, although a global infection, *C. gattii* is also predominantly associated with arboreal tree species. It is hypothesised that *C. gattii*’s spread to the Pacific Northwest may have been through shipping ballast combined with ocean currents and perhaps aided by extreme events such as Tsunami (Engelthaler and Casadevall 2019), and/or via the plant and seed trade (Roe *et al*. 2018).

How the biotic and abiotic environment shapes exposure and epidemiology of cryptococcosis at a more local level is yet to be determined. Environmental surveillance in Zambia has suggested an ecological split between *C. neoformans* which was found mostly in the southern, arid and low altitude Zambezi Mopane ecoregion, and *C. gattii* in the northern, wet and high altitude Central Miombo ecoregion (Vanhove *et al*. 2017). This ecological divide could be significant if it affects the distribution of clinical cases and the relative risk of exposure, particularly among populations of HIV-infected individuals that inhabit each part of the country (Maziarz and Perfect 2016). Further environmental sampling and enhanced clinical diagnosis to distinguish, at minimum, the infectious agent at the level of the species complex could disentangle the effect of geographic species distribution on clinical incidence at sub-national levels. This could ultimately affect recommendations given to health service providers on diagnosis and drug stewardship upon presentation of a case of pneumonia or meningitis, as well as the utility of prophylaxis, if the risk of local acquisition of *C. neoformans* infection is high (Oladele *et al*. 2017).

It is highly likely that not all environmental niches of *Cryptococcus* have as yet been identified. This was recently demonstrated by the discovery of VGV from investigative sampling of an entirely new ecological niche, the rock hyrax midden, where it was found to co-exist with other cryptococcal molecular types (Farrer *et al*. 2019). Hyrax middens are extremely stable and long-lasting structures that can exist in the same place for thousands of years (Chase *et al*. 2012). Middens have a high nitrogen content which is known to aid cryptococcal growth, which likely results in the development of patchy high-burden hotspots of *Cryptococcus*. Twinned with their extreme environmental stability, hyrax middens may therefore provide stable long-term evolutionary arenas that are important in generating diversity of *Cryptococcus* (Staib *et al*. 1978; Vreulink *et al*. 2020).

How each identified niche relates to being a reservoir of infection and hence when and where people are exposed remains unclear. In California, USA, isolation of VGIII environmental isolates showed a very close relationship with clinical isolates suggesting a local environmental reservoir of infection (Springer *et al*. 2014), and similar studies are ongoing in the UK (Farrer *et al*.). However, as yet the genomic epidemiology to explore these links have not been made in Africa. Since VNI is found frequently around the globe in pigeon faeces from urban locations, it is easy to anticipate how people may be exposed to VNI more often, thereby leading to more frequent infection. Yet many observed (and more diverse) ecological niches are found in very rural locations far from human activity and thus may not pose an immediate clinical threat through exposure. Conversely, some clinically significant molecular types, such as VNII, are rarely found in the environment and thus their infectious reservoir is, as yet, unknown.

**Figure 4.**
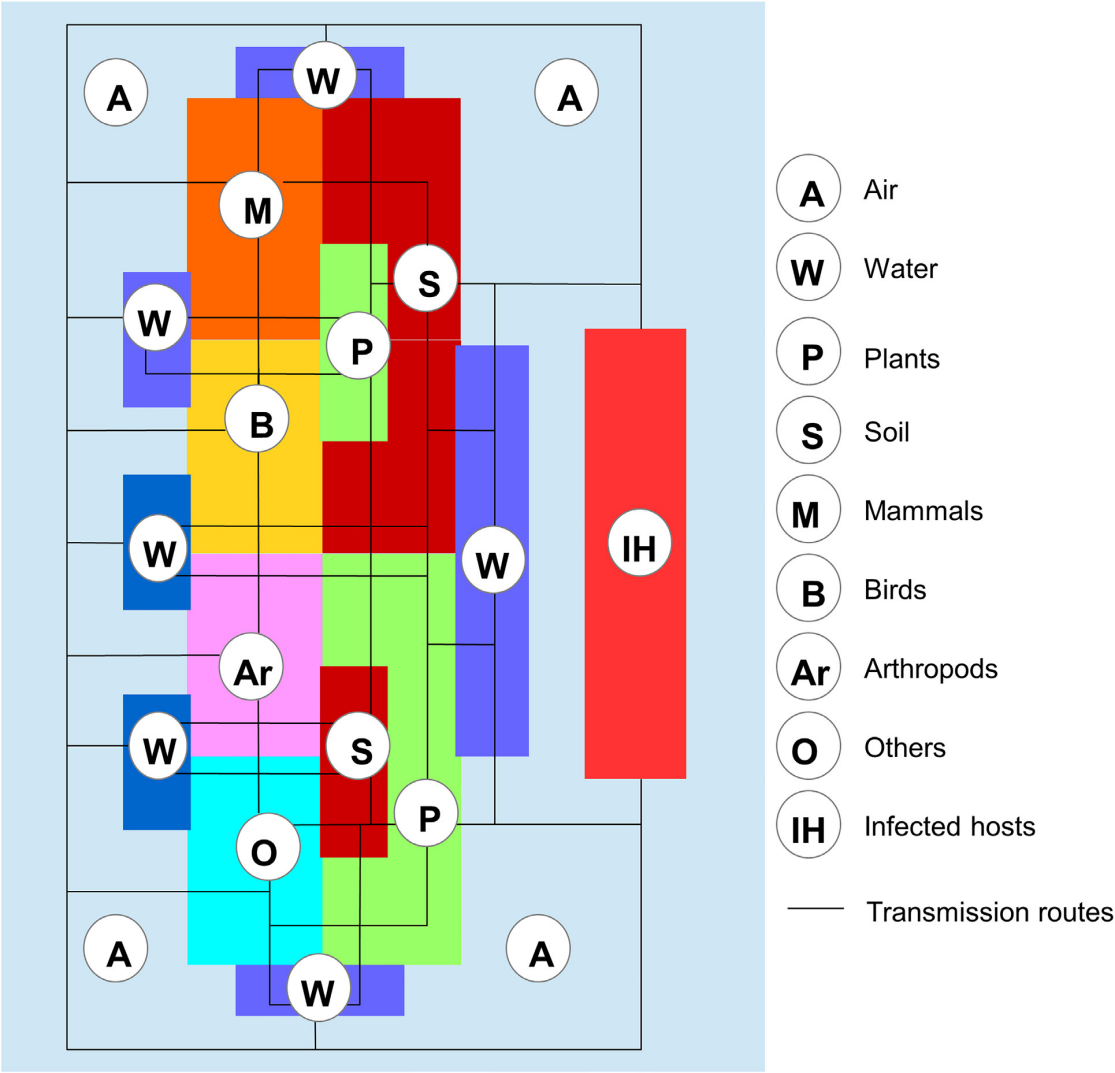
Schematic representation of the relationships among biotic and abiotic components (coloured polygons) of the *Cryptococcus* ecosystem, and dynamic flow of the fungus through the different niches (solid lines). *Cryptococcus* can circulate in the environment through several vectors (wind, water, animals) and reach the main reservoirs (soil and plants). From its habitat in these reservoirs, *Cryptococcus* can produce and release aerosolised basidiospores which are able to colonize other niches or infect susceptible hosts.

The interaction of *Cryptococcus* with its environment and susceptible hosts is complex (Fig. 4). Although *Cryptococcus* spp. are environmental saprotrophs, thriving on decaying wood, soil and animal droppings, they can also be found in water, including ocean saltwater (Emmons 1955; Kidd *et al*. 2007b; Kandasamy, Alikunhi and Subramanian 2012). *Cryptococcus* is likely actively dispersed between sites through contamination of a variety of animal species that live or feed on colonised trees or soil, including insects (23). Passive dispersal occurs through the production and aerosolization of desiccated yeast cells or through basidiospores that are produced during sexual reproduction (Zhao *et al*. 2019; Cogliati *et al*. 2020). These cells and spores may disperse widely before colonising new habitats, and are thought to represent the principle exposure to susceptible hosts through inhalation (Velagapudi *et al*. 2009; Rieux *et al*. 2014).

Since trees have been shown to be one of the main reservoirs for cryptococcal yeasts, understanding the biotic and abiotic components that comprise these tree-scale ecological niches alongside which vectors contribute to the spread of *Cryptococcus* in the environment could aid understanding of the mechanisms involved in human infection. In a recent study, biotic and abiotic factors affecting the distribution of both *C. neoformans var. neoformans* and *C. neoformans var. grubii* found living on the same oak tree were investigated (Cogliati *et al*. 2020). Ants and other arthropods were shown to contribute to the distribution of the yeasts on the tree as well as to the colonisation of other trees. Microscopy showed how the yeast cells use filamentous protrusions to anchor to the bark, leaving the non-adherent surface free for budding, the resulting spores of which were identified in the surrounding air. These studies may implicate arthropods as important hosts for *Cryptococcus*, and may in part explain the utility of the wax-moth larvae *Galleria mellonella* as a model for cryptococcal virulence (Mylonakis *et al*. 2005).

Although infection is caused by aerosolised infectious propagules, airborne isolations of *Cryptococcus* are scarce. Most attempts to isolate cryptococcal spores have simply exposed agar Petri dishes to the air, a few of which have been successful, mostly when plates are exposed directly next to pigeon guano sources or when spores have been aerosolized through human intervention (Baroni *et al*. 2006; Randhawa *et al*. 2006; Pedroso, Ferreira and Candido 2009). Other attempts have been made with high-throughput air samplers to trap *Cryptococcus* bioaerosols (Lazera *et al*. 2000; Kidd *et al*. 2007b, 2007a). Use of high-throughput air sampling in Canada found that forestry activities led to a higher concentration of *C. gattii* spores in the air (Kidd *et al*. 2007a). This may be relevant to exposure risk in southern Africa since mopane trees, which are strongly associated with colonisation by *C. neoformans* (Litvintseva *et al*. 2011; Vanhove *et al*. 2017), form an important part of the local culture and are frequently cut and used for charcoal, traditional medicine, building materials and the cultivation of edible mopane worms (Chidumayo 1993; Woollen *et al*. 2016; Ziba and Grouwels 2017). Seasonality may also affect the concentration of infectious propagules released into the air, with autumn conditions associated with a greater concentration of airborne cryptococcal propagules observed in the temperate climate of northern Italy (Cogliati *et al*. 2020).

An added complication in assessing from where and when infection occurs is the hypothesis that infection may occur many months-to-years before symptoms. A study by Beale *et al*. (Beale *et al*. 2015) found a lack of geographic clustering between genetic sequences from patients in Cape Town, suggesting against local acquisition of infection, though the study did not attempt to support this with surveillance of the environment. Combining clinical genetic studies such as this with environmental surveillance around people's houses and in line with their travel and activity history (particularly activities related to forestry), may give more insights into from where and when infection is acquired.

## EMERGENCE OF ANTIFUNGAL DRUG RESISTANCE

Treatment failure and subsequent relapse of infection can occur as a result of cryptococcal resistance to first-line drug treatment, including azoles and flucytosine (FLC) (Birley *et al*. 1995; Aller *et al*. 2000; Musubire 2013; Billmyre *et al*. 2020). Development of resistance and emergence of heteroresistant colonies is apparent in serially collected isolates from patients and relapse patients, suggesting resistance can develop as a within-host response to drug treatment (Chen *et al*. 2017; Stone *et al*. 2019). In some clinical cases, nonsense mutations in the gene encoding DNA mismatch repair proteins (*MSH2*,  *MSH5*, *RAD5 and POL3*) are associated with hypermutator phenotypes that can lead to very rapid within-host microevolution (Rhodes *et al*. 2017a; Boyce *et al*. 2020). When twinned with drug-pressure, hypermutating genotypes are associated with the emergence of drug-resistance *in vitro* and present a novel pathway for rapid evolution of resistance to first-line antifungal drugs (Boyce *et al*. 2017). The relevance of hypermutators in the environmental stages of *Cryptococcus* has not been established, however. Differing levels of resistance to antifungals have been identified in environmental isolates suggesting that either hypermutator or other, perhaps innate, resistance mechanisms may be ecologically relevant. For instance, sampling in Cameroon found both *C. neoformans* and *C. gattii* in pigeon and bat guano with high antifungal resistance (Dongmo *et al*. 2016). In another region of Africa, both environmental VGIV / VGV strains from Zambia showed unusually high resistance to flucytosine (FLC), and in particular isolates from a specific clade of VGV (VGV-A) (Farrer *et al*. 2019). The ability of environmental isolates to manifest resistance to first-line drugs could either be the indirect consequence of adaptation to antifungal-like chemicals in the environment or the direct consequence of exposure to fungicides (such as azoles) that are used in agriculture or forestry. Evidence that azole resistance works at least partly through upregulation of ABC transporters which act to remove molecules from cells in a non-specific manner suggests the former may be true (Posteraro *et al*. 2003; Sanguinetti *et al*. 2006). Of relevance, there is widespread concern that widespread use of azoles in agriculture and forestry industries is contributing to emerging resistance in other fungi, most notably *Aspergillus fumigatus* (Snelders *et al*. 2012; Chowdhary *et al*. 2013; Kleinkauf *et al*. 2013; Ren *et al*. 2017). Surveillance of resistance in environmental cryptococcal populations may well be important to monitor the emergence and spread of resistance and thus the threat to clinical management of disease. Mapping environmental isolations against areas of intensive farming and commercial forestry may also indicate whether there is an effect of azole usage on propagating these genotypes by creating hotspots for the evolution of antifungal resistance.

## CHALLENGES IN ENVIRONMENTAL SURVEYING AND MODELLING CRYPTOCOCCAL DISTRIBUTIONS

It is clear there is much to learn from the genomics of environmental cryptococcal populations. However, isolating *Cryptococcus* spp. from the environment is challenging—it can be difficult to find and, once found, can be problematic to isolate into pure culture due to competition from faster-growing filamentous fungi (Lazera *et al*. 2000; Pham *et al*. 2014; de Matos Castro e Silva *et al*. 2015). Surveying and subsequent culturing can thus be labour and time-intensive and results in limited recovery rates (Vilcins *et al*. 2002; Kidd *et al*. 2007b; Litvintseva *et al*. 2011; Cogliati *et al*. 2016; Vanhove *et al*. 2017). Because of this and the propensity to find *Cryptococcus spp*. in certain ecological niches, targeted sampling should be used in order to generate a larger number of isolates for study. However, targeted sampling leads to issues if using data to conduct environmental niche modelling (ENM) due to positive selection bias (Mak *et al*. 2010; Cogliati *et al*. 2017; Vanhove *et al*. 2017; Alaniz *et al*. 2020). ENM studies attempt to map the distribution of *Cryptococcus* spp. across entire countries or continents using climatic variables highly dependent on a small number of sampled collection sites. Models have focused on use of presence-only data since absence of the pathogen from locations that may not have been sampled cannot be assumed and negatively sampled locations may not indicate true absence since the yeast may just not have been recovered successfully in culture. Presence-absence models perform better than presence-only models but models for wide-ranging and tolerant species can be particularly sensitive to absence data, as has been shown in predictions of bird habitats (Brotons *et al*. 2004; Elith *et al*. 2006). Use of pseudo-absence data has been proposed as a potential strategy in such situations (Gu and Swihart 2004) (Gu and Swihart 2004) (108) (108) (108) (Zaniewski, Lehmann and Overton 2002; Engler, Guisan and Rechsteiner 2004; Gu and Swihart 2004; Phillips *et al*. 2009; Lobo, Jiménez-Valverde and Hortal 2010; Senay, Worner and Ikeda 2013). Since different species distribution models also show differences in predictive performance and stability, different algorithms should be compared to give an indication of uncertainty between methods, in a process that is analogous to the use of climatic ensemble models (Ren-Yan *et al*. 2014).

## CONCLUSION

Despite insights into the ecology, biology, evolution and epidemiology that environmental isolates of *C. neoformans/C. gattii* provide, sampling and subsequent genomic and phenotypic analysis of environmental isolates have, to date, been limited, particularly within the African context. This is despite recent progress stemming from both ecological surveys and genomic epidemiology showing that we are underestimating the scale and clinical importance of cryptococcal diversity. While increased sampling and genomics analysis of *Cryptococcus* in the southern Africa region would be of benefit since this region appears to be the origin of global diversity and has the highest clinical impact, sampling has been very limited in other regions, particularly Central Africa which may be important given that it borders the southern region. Although here we have focused on *Cryptococcus*, the significance of the methods and analyses we describe are applicable to other environmental fungi and microbes that pose an increasing threat to human, animal and plant health and biosecurity (Fisher *et al*. 2012). The integration of data from multiple sources, including environmental, clinical, bioclimatic, molecular and epidemiological, is becoming increasingly important in understanding the complexity of microbial threats. Indeed, the integrative environment-health science frameworks that we describe here are increasingly needed to understand and model future scenarios with the aim of thwarting future outbreaks of infection (Fisher and Murray 2021).

## Supplementary Material

fiab093_Supplemental_File
