# Supplementary material for: The need for environmental surveillance to understand the ecology, epidemiology and impact of Cryptococcus infection in Africa
Source: FEMS Microbiol Ecol. 2021 Jul 1;97(7):fiab093. doi: 10.1093/femsec/fiab093 (PMC8536938; doi:10.1093/femsec/fiab093)
Supplement: fiab093_Supplemental_File [file fiab093_supplemental_file.doc]

Supplementary Table. Clinical *Cryptococcus neoformans* and *Cryptococcus gattii* cases/isolates reported in Africa

| Country  (Total isolates) | Place of isolation | Number of isolates | Species, variety, serotype, molecular type | reference |
| --- | --- | --- | --- | --- |
| Morocco  (97) | Rabat  Rabat  Rabat  Rabat  Casablanca  Rabat  Rabat | 1  2  9  40  43  1  1 | *C. neoformans/C. gattii*  *C. neoformans/C. gattii*  *C. neoformans/C. gattii*  *C. neoformans/C. gattii*  *C. neoformans/C. gattii*  *C. neoformans/C. gattii*  *C. neoformans/C. gattii* | Houda et al. Pan Afr Med J. 2011; 8:42  Lamzaf et al. J Fr Ophtalmol. 2011;34(2):75-82  Aoufi et al. Ann Biol Clin (Paris). 2008. 66(1):79-81  Bandadi et al. Pan Afr Med J. 2019;33:249  Dollo et al. J Mycol Med. 2016;26(4):331-336  Elkhihal et al. J Mycol Med. 2015;25(3):208-12  Hajoui et al. Med Sante Trop. 2014;24(3):317-9 |
|
|
| Tunisia  (3) | Tunis  Monastir  Tunis | 1  1  1 | *C. neoformans/C. gattii*  *C. neoformans* species complex  *C. neoformans/C. gattii* | Nfoussi et al. Med Trop (Mars). 2010;70(1):85-7  Aloui et al. Saudi J Kidney Dis Transpl. 2013;24(1):72-75  Beji et al. Saudi J Kidney Dis Transpl. 2017;28(6):1435-1439 |
| Egypt  (55) | Cairo  Cairo  Zagazig  Zagazig  Cairo | 10  1  29  7  3  5 | *C. neoformans* var. *grubii*, A  *C. gattii* species complex  *C. neoformans* species complex  *C. neoformans* var. *grubii*, A  *C. neoformans*, AD hybrids  *C. neoformans/C. gattii* | Elias et al. J Egypt Public Health Assoc. 2009;84(1-2):169-81  Mansour et al. East Mediterr Health J. 2006;12(1-2):241-4  Abdel-Salam. Mycoses. 2005;48(5)  Abdel-Salam. Folia Microbiol 2003;48:261  Soliman et al. Trans R Soc Trop Med Hyg. 1995;89(4):410 |
|
|
|
|
| Libya  (2) | Tripoli | 2 | *C. neoformans* var. *grubii*, VNI | Ellabib et al. J Mycol Medicale. 2017;27(3):421-424 |
| Algeria  (4) | Algiers | 4 | *C. neoformans/C. gattii* | Karaouzene et al. Arch Inst Pasteur Alger. 1998;62:192-9 |
| Mali  (34) | Bamako  Bamako  Bamako | 14  3  17 | *C. neoformans/C. gattii*  *C. neoformans/C. gattii*  *C. neoformans/C. gattii* | Minta et al. Med Trop (Mars). 2011;71(6):591-5  Minta et al. Bulll Soc Pathol Exot. 2008;101(4):308-10  Oumar et al. Rev Med Brux. 2008;29(3):149-52 |
|
|
| Gambia  (2) | Katabas | 2 | *C. neoformans/C. gattii* | Wakefield et al. Trans R Soc Trop Med Hyg. 1990;84(6):800-2 |
| Guinea Bissau  (20) | Bissau | 20 | *C. neoformans/C. gattii* | Thomsen et al. Pan Afr Med J. 2018;29:18 |
| Guinea  (49) | Conakry | 49 | *C. neoformans/C. gattii* | Traoré et al. Med Sante Trop. 2015;25(1):52-5 |
| Ethiopia  (189) | Addis Ababa  Addis Ababa  Addis Ababa  Gondar  Addis Ababa  Addis Ababa  Oromia region  Addis Ababa  Adama  Addis Ababa  Asella and Adama  Bahir Dar | 31  19  1  9  1  31  26  8  2  18  27  16 | *C. neoformans/C. gattii*  *C. neoformans/C. gattii*  *C. neoformans/C. gattii*  *C. neoformans/C. gattii*  *C. neoformans/C. gattii*  *C. neoformans/C. gattii*  *C. neoformans/C. gattii*  *C. neoformans/C. gattii*  *C. neoformans/C. gattii*  *C. neoformans/C. gattii*  *C. neoformans/C. gattii*  *C. neoformans/C. gattii* | Seboxa et al. Ethiop Med J. 2010;48(3):237-41  Woldemanuel & Haile. Ethiop Med J. 2001;39(3):185-92  Gebremedhin. Ethiop Med J. 1992;30(3):169-73  Aseffa et al. J Infect. 1997;35(3):323-4  Mengistu et al. Ethiop Med J. 2011;49(4):349-59  Alemu et al. PLOS ONE. 2013;8(3):e58377  Beyene et al.PLOS ONE. 2013;8(10):e75585  Mihret et al. Ethiop Med J. 2014;Suppl 1:43-8  Reepalu et al. BMC Res Notes. 2015;8:702  Mamuye et al. Am J Trop Med Hyg. 2016;95(4):789-792  Beyene et al. Clin Inf Dis. 2017;65(12):2126-2129  Derbie et al. Ethiop J Health Sci. 2018;28(4):369-374 |
| Senegal  (116) | Dakar  Dakar  Senegal  Dakar  Dakar  Dakar | 3  1  1  1  106  4 | *C. neoformans/C. gattii*  *C. neoformans/C. gattii*  *C. gattii*, B, VGII  *C. neoformans/C. gattii*  *C. neoformans/C. gattii*  *C. neoformans/C. gattii* | Ndiaye et al. Med Trop (Mars). 2011;71(2):176-8  Ndiaye et al. Arch Pediatr. 2010;17(7):1069-71  Fraser et al. Nature. 2005;437(7063):1360-1364  Seydi et al. Dakar Med. 2001;46(1):65-7  Sow et al. Mycopathologia. 2013;176(5-6):443-9  Soumare et al. Med Trop (Mars). 2005;65(6):559-62 |
| Cameroon  (384) | Cameroon  Yaoundé  Yaoundé  Yaoundé  Yaoundé  Yaoundé  Yaoundé  Yaoundé | 2  114  29  41  33  150  14  1 | *C. gattii*, B  *C. neoformans* var*. grubii*, VNI  *C. neoformans/C. gattii*  *C. neoformans/C. gattii*  *C. neoformans/C. gattii*  *C. neoformans* var*. grubii*, VNI  *C. neoformans/C. gattii*  *C. neoformans/C. gattii* | Kwon-Chung & Bennett. Am J Epidemiol. 1984;120(1):123-30  Bertout et al. Clin Microbiol Infect. 2013;19:763-769  Dzoyem et al. Afr Health Sci. 2012;12(2):129-33  Kammalac Ngouana et al. J Mycol Med. 2015;25(1):11-6  Mbuagbaw et al. Afr J Neurol Sci. 2006;25(2)  Kammalac Ngouana et al. J Med Microbiol. 2015;65(7)  Temfack et al. Front Microbiol. 2018;9:409  Essouma et al. J Med Case Rep. 2019;13(1):86 |
| Ivory Coast  (768) | Abidjan  Abidjan  Abidjan  Ivory Coast  Abidjan  Abidjan  Ivory Coast  Treichville d’Abijan  Abijan  Ivory Coast  Ivory Coast | 2  2  44  5  70  61  1  22  4  40  312  6  32  13  5  149 | *C. neoformans/C. gattii*  *C. neoformans/C. gattii*  *C. neoformans/C. gattii*  *C. neoformans* var. *grubii*, A  *C. neoformans/C. gattii*  *C. neoformans/C. gattii*  *C. gattii*, B  *C. neoformans/C. gattii*  *C. gattii*, B, VGII  *C. neoformans,* AD hybrids, VNIII  *C. neoformans* var*. grubii*, VNI  *C. neoformans* var*. grubii*, VNII  *C. neoformans* var*. grubii*, VNI  *C. neoformans,* AD hybrids, VNIII  *C. gattii*, B, VGII  *C. neoformans/C. gattii* | Adonis-Koffy et al. Arch Pediatr. 2010;17(7):1072-3  Kouame-Assouan et al. Bull Soc Pathol Exot. 2007;100(1):15-6  Ouedraogo et al. Mali Med. 2007;22(1):26-8  Chandenier et al. Eur J Clin Microbiol Infect Dis 2004;23(6):506-8  Eholie et al. Bull Soc Pathol Exot. 2000;93(1):50-4  Eholie et al. Bull Soc Pathol Exot. 1997;90(5):307-11  Kwon-Chung, Bennett. Am J Epidemiol. 1984;120(1):123-30  Kadjo et al. J Mycol Med. 2011;21(1):6-9  Kassi et al. Mycoses. 2016;59(12)  Kassi et al. J Med Microbiol. 2018;67(1):87-96  Bissangnene et al. Med Mal Infect. 1994;24:580-5 |
| Burkina Faso  (50) | Bobo-Dioulasso  Bobo-Dioulasso  Bobo-Dioulasso  Bobo-Dioulasso | 36  8  5  1 | *C. neoformans/C. gattii*  *C. neoformans/C. gattii*  *C. neoformans/C. gattii*  *C. neoformans/C. gattii* | Millogo et al. Bull Soc Pathol Exot. 2004;97(2):119-21  Millogo et al. Bull Soc Pathol Exot. 1999;92(1):23-6  Ki-Zerbo et al. Med Trop (Mars). 1996;56(1):63-5  Béogo et al. J Mycol Med. 2014;24(4) |
| Benin  (6) | Cotonou | 6 | *C. neoformans/C. gattii* | Ogouyémi-Hounto et al. J Mycol Med. 2016;26(4):391-397 |
| Ghana  (24) | Kumasi  Acra  Kumasi | 2  3  19 | *C. neoformans/C. gattii*  *C. neoformans/C. gattii*  *C. neoformans/C. gattii* | Mamoojee et al. Trop Med Int Health. 2011;16(1):53-6  Opintan et al. Trans R Soc Trop Med Hyg. 2017;111(10):464-471  Owusu et al. Ann Clin Microbiol Antimicrob. 2012;11:28 |
| Nigeria  (220) | Benin City  Llorin  Port Harcourt  Nigeria  Nnewi  Nigeria | 150  1  1  1  4  63 | *C. neoformans/C. gattii*  *C. neoformans/C. gattii*  *C. neoformans/C. gattii*  *C. neoformans/C. gattii*  *C. neoformans/C. gattii*  *C. neoformans/C. gattii* | Osazuwa et al. Oman Med J. 2012;27(3):228-31  Salami et al. West Afr J Med. 2009;28(5):343-6  Eghwrudjakpor & Allison. Acta Neurochir (Wien). 2009;151(6):711-2  Ashiru & Akang. Mycopathologia. 1994;127(1):15-7  Chukwuanukwu et al. J Infect Public Health. 2019;13(7):1042-1046  Ezeanolue et al. J Acquir Defic Syndr. 2016;73(1):117-21 |
| Equatorial Guinea (18) | Bata | 18 | *C. neoformans/C. gattii* | Wang & Carm. Trop Doct. 2001;31(4):221-2 |
| Gabon  (20) | Libreville  Gabon | 11  9 | *C. neoformans/C. gattii*  *C. neoformans* var. *grubii*, A | Ondounda et al. Med Trop (Mars). 2010;70(4):406  Chandenier et al. Eur J Clin Microbiol Infect Dis 2004;23(6):506-8 |
| Democratic Republic of Congo (130) | Zaire  Zaire  Kinshasa  Zaire  Kinshasa  Zaire  Kinshasa  Kinshasa  Zaire  Zaire  Zaire  Kinshasa  Zaire  Zaire | 3  4  1  1  8  1  1  7  6  29  1  41  6  1  14  3  2  1 | *C. neoformans* var*. grubii,*A, VNI  *C. gattii*, B, VGI  *C. gattii*, B  *C. neoformans* species complex  *C. neoformans* var*. grubii,*A  *C. gattii*, B  *C. gattii* species complex  *C. neoformans* var*. grubii,* A  *C. neoformans/C. gattii*  *C. neoformans/C. gattii*  *C. gattii* species complex  *C. neoformans* species complex  *C. gattii* species complex  *C. neoformans/C. gattii*  *C. neoformans/C. gattii*  *C. neoformans* var*. grubii,* A  *C. neoformans* var*. grubii,* A, VNI  *C. gattii*, B | Boekhout et al. Microbiol. 2001;147(Pt 4):891-907  Xu et al. Mol Ecol. 2000;9(10):1471-81  Varma et al. J Clin Microbiol. 1995;33(7):1807-1814  Dromer et al. J Clin Microbiol. 1993;31(2):359-363  Muyembe et al. Med Trop (Mars) 1992;52:435-438  Polacheck et al. J Clin Microbiol. 1992;30(4):925-30  Perriens et al. J Acquir Immune Defic Syndr. 1992;5:333-340  Desmet et al. AIDS. 1989;3(2):77-8  Kapend’a et al. Eur J Clin Microbiol Infect Dis. 1987;6(3):320-321  Swinne et al. Ann Soc Belg Med Trop. 1986;66(1):57-61  Disengomoka et al. Ann Pediatr (Paris). 1983;30(2):127-30  Lontie et al. Ann Soc Belg Med Trop. 1973;53(6):619-32  Litvintseva et al. Genetics. 2006;172(4):2223-2238  Kwon-Chung & Bennett. Am J Epidemiol. 1984;120(1):123-30 |
| Congo  (20) | Congo  Brazaville | 19  1 | *C. neoformans* var*. grubii,* A  *C. neoformans/C. gattii* | Chandenier et al. Eur J Clin Microbiol Infect Dis 2004;23(6):506-8  Molez et al. Med Trop (Mars). 1982;42(5):561-3 |
| Uganda  (3056) | Kampala  Kampala  Kampala  Entebbe  Kampala  Kampala  Kampala  Uganda  Mbarara  Mbarara  Kampala  Kampala  Kampala  Kampala  Kampala  Kampala  Kampala  Uganda  Kampala  Kampala  Kampala and Mbarara  Kampala  Uganda  Kampala and Mbarara  Kampala | 24  1  15  77  120  43  69  38  8  10  5  2  60  30  78  1  1  138  1  98  1  94  121  605  5  696  127  5  571  12 | *C. neoformans/C. gattii*  *C. neoformans/C. gattii*  *C. neoformans/C. gattii*  *C. neoformans/C. gattii*  *C. neoformans/C. gattii*  *C. neoformans/C. gattii*  *C. neoformans* var*. grubii,* A, VNI  *C. neoformans* var*. grubii,* A, VNII  *C. neoformans*, AD hybrids, VNIII  *C. neoformans* var*. grubii,* A  *C. neoformans* var*. grubii,* A, VNI  *C. neoformans* var*. grubii,* A, VNII  *C. neoformans/C. gattii*  *C. neoformans/C. gattii*  *C. neoformans/C. gattii*  *C. neoformans/C. gattii*  *C. neoformans/C. gattii*  *C. neoformans/C. gattii*  *C. neoformans/C. gattii*  *C. neoformans* var*. grubii,* A, VNI  *C. neoformans/C. gattii*  *C. neoformans/C. gattii*  *C. neoformans/C. gattii*  *C. neoformans/C. gattii*  *C. neoformans/C. gattii*  *C. neoformans/C. gattii*  *C. neoformans* var*. grubii,* A, VNI  *C. neoformans*, AD hybrids, VNIII  *C. neoformans/C. gattii*  *C. neoformans/C. gattii* | Oyella et al. J Int AIDS Soc. 2012;15:15  Natukunda et al. AIDS Res Hum Retrovir. 2011;27(4):373-376  Yoo et al.J Acquir Immune Defic Syndr. 2010;54(3):269-274  French et al. AIDS. 2002;16(7):1031-8  Pfaller et al. Diagn Microbiol Infect Dis. 1998;32(3):191-9  Davey et al. J Antimicrob Chemother. 1998;42(2):217-220  Wiesner et al. mBio. 2012;3(5):e00196-12  Litvintseva et al. Genetics. 2006;172(4):2223-2238  Longley et al. Clin Inf Dis. 2008;47(12):1556-1561  Muzoora et al. J Infect. 2012;64(1):76-81  Carlson et al. Metab Brain Dis. 2014;29(2):269-279  Kiggundu et al. Med Mycol Case Rep. 2014;5:16-9  Velamakanni et al. Med Mycol Case Rep. 2014;6:10-3  Williams et al. Clin Inf Dis. 2015;61(3):464-467  Musubire et al. Med Mycol Case Rep. 2015;8:40-3  Smith et al. Antimicrob Agents Chmother. 2015;59(12):7197-7204  Mpoza et al. Med Mycol Case Rep. 2017;19:30-32  Nalintya et al. J Acquir Immune Defic Syndr. 2018;78(2):231-238  Diehl et al. Open Forum Infect Dis. 2018;5(6):ofy105  Rhein et al. Open Forum Infect Dis. 2018;5(8):ofy122  Ssebambulidde et al. Clin Infect Dis. 2019;68(12):2094-2098  Atherton et al. Wellcome Open Res. 2018;3:80  Ashton et al. Nature Comm 2019;10:2035  Ellis et al. Open Forum Infect Dis. 2019;6(10):ofz419  Pastick et al. Med Mycol. 2020;58(3):282-292 |
|
| Kenya  (361) | Nairobi  Nairobi  Nairobi  Nairobi  Nairobi  Kenya  Nyanza province  Chogoria  Nairobi  Bungoma county | 63  4  75  1  2  2  76  4  1  2  59  1  64  2  4  1 | *C. neoformans* species complex  *C. gattii* species complex  *C. neoformans* var*. grubii,* A  *C. neoformans* var*. neoformans,* D  *C. neoformans*, AD hybrids  *C. gattii* species complex  *C. neoformans/C. gattii*  *C. neoformans/C. gattii*  *C. neoformans/C. gattii*  *C. gattii,* B  *C. neoformans/C. gattii*  *C. neoformans/C. gattii*  *C. neoformans* var. *grubii*, VNI  *C. neoformans* var. *grubii*, VNII  *C. gattii,* VGI  *C. neoformans/C. gattii* | Mdodo et al. Mycoses. 2011;54(5)  Bii et al. Mycoses. 2006;50(1)  Odhiambo et al. East Afr Med J. 1997;74(9):576-8  Wanyoike et al. East Afr Med J. 1995;72(10):658-60  Joshi et al. East Afr Med J. 1989;66(1):69-73  Kwon-Chung & Bennett. Am J Epidemiol. 1984;120(1):123-30  Meyer et al. Trop Med Int Health. 2013;18(4):495-503  O’Reilly. Paediatr Int Child Health. 2016;36(2):154-6  Kangogo et al. Mycoses. 2015;58(11)  Owour & Chege. BMC Infect Dis. 2019;19(1) |
| Rwanda  (533) | Rwanda  Rwanda  Kigali  Kigali  Rwanda  Kigali  Rwanda | 19  2  1  491  8  5  3  1  2  1 | *C. neoformans* var. *grubii*, A  *C. neoformans* var. *grubii*, A, VNI  *C. neoformans* var. *grubii*, A, VNB  *C. neoformans* species complex  *C. gattii* species complex  *C. neoformans/C. gattii*  *C. gattii,* B  *C. gattii,* C  *C. gattii* species complex  *C. neoformans/C. gattii* | Chandenier et al. Eur J Clin Microbiol Infect Dis 2004;23(6):506-8  Boekhout et al. Microbiol. 2001;147(Pt 4):891-907  Bogaerts et al. J Infect. 1999;39(1):32-7  Mukamurangwa et al. Bull Soc Pathol Exot. 1994;87(1):41-4  Dromer et al. J Clin Microbiol. 1993;31(2):359-363  Bogaerts et al. Trans R Soc Trop Med Hyg. 1993;87(1):63-64  Rogerie et al. Med Trop (Mars). 1987;47(2):185-8 |
| Zambia  (182) | Lusaka  Lusaka  Lusaka  Lusaka | 64  16  3  4  13  82 | *C. neoformans/C. gattii*  *C. neoformans* var. *grubii*, VNI  *C. neoformans* var. *grubii*, VNII  *C. neoformans* var. *grubii*, VNB  *C. neoformans/C. gattii*  *C. neoformans/C. gattii* | Siddiqi et al. Clin Infect Dis. 2014;58(12):1771-1777  Vanhove et al. Mol Ecol. 2017;26(7):1991-2005  Siddiqi et al. Neurology. 2017;88(5):477-482  Haachambwa et al. Open Forum Infect Dis. 2019;6(10):ofz336 |
| Namibia  (26) | Windhoek | 26 | *C. neoformans/C. gattii* | Sawadogo et al. PLOS ONE. 2016;11(10):e0161830 |
| Madagascar  (13) | Antananarivo and Toamasina | 3  4  6 | *C. neoformans* var. *grubii*, VNI  *C. neoformans* var. *grubii*, VNII  *C. neoformans* v. *grubii*, VNI/VNII | Rakotoarivelo et al. PLOS Negl Trop Dis. 2020;14(1):e0007984 |
| Lesotho  (14) | Lesotho | 14 | *C. neoformans/C. gattii* | Rick et al. PLOS ONE. 2017;12(9):e0183656 |
| Burundi  (3) | Bujumbura  Bujumbura | 2  1 | *C. neoformans* species complex  *C. neoformans/C. gattii* | Varma et al. J Clin Microbiol. 1995;33(7):1807-1814  Niyongabo et al. Med Trop (Mars). 1992;52(2):179-81 |
| Tanzania  (333) | Mwanza  Dar es Salama  Tanzania  Tanzania  Moshi  Mwanza  Mwanza  Mwanza  Mwanza  Tanzania | 15  175  10  4  1  7  10  1  14  1  95 | *C. neoformans/C. gattii*  *C. neoformans/C. gattii*  *C. neoformans* var*. grubii,* A  *C. neoformans* var*. grubii,* A, VNI  *C. neoformans* var*. grubii,* A  *C. neoformans/C. gattii*  *C. neoformans/C. gattii*  *C. neoformans/C. gattii*  *C. neoformans/C. gattii*  *C. gattii,* VGII  *C. neoformans/C. gattii* | Wajanga et al. J Int AIDS Soc. 2011;14:48  Matee & Matre. East Afr Med J. 2001;78(9):458-60  Litvintseva et al. Genetics. 2006;172(4):2223-2238  Xu et al. Mol Ecol. 2000;9(10):1471-81  Rugemella et al. Trop Med Int Health. 2013;18(9):1075-1079  Magambo et al. J Int AIDS Soc. 2014;17(1):19040  Gunda et al. Clin Case Rep. 2015;3(9)  Boaz et al. J Trop Med. 2016;2016:6573672  Wilson et al. Med Mycol Case Rep. 2018;22:4-7  Kisenge et al. BMC Infect Dis. 2007;7:39 |
| Malawi  (200) | Zomba  Lilongwe  Malawi  Blantyre  Blantyre  Lilongwe, Blantyre  Blantyre | 1  8  4  2  1  16  133  3  31  1 | *C. neoformans/C. gattii*  *C. neoformans* var*. grubii,* A  *C. neoformans* var*. grubii,* A, VNI  *C. gattii,* C, VGIV  *C. neoformans*, AD hybrids  *C. neoformans/C. gattii*  *C. neoformans/C. gattii*  *C. neoformans/C. gattii*  *C. neoformans/C. gattii*  *C. neoformans/C. gattii* | Kumwenda et al. Malawi Med J. 2010;22(2):57-58  Litvintseva et al. J Infect Dis. 2015;192(5):888-892, Bell et al. Int J Infect Dis. 2001;5(2):63-9, Litvintseva et al. Genetics. 2006;172(4):2223-2238  Archibald et al. Emerg Infect Dis. 2014;10(1):143-5  Gordon et al. Clin Infect Dis. 2000;31(1):53-7  Subramanyam et al. Ann Trop Paediatr. 1997;17(2):165-7  Maher & Mwandumba. J Infect. 1994;28(1):59-64  Ellis et al. BMC Infect Dis. 2018;18(520) |
| Zimbabwe  (138) | Harare  Harare  Zimbabwe  Harare  Harare  Harare  Harare  Harare | 13  3  8  16  2  28  40  15  9  3  1 | *C. neoformans/C. gattii*  *C. neoformans/C. gattii*  *C. neoformans* var*. grubii,* A, VNI  *C. neoformans/C. gattii*  *C. neoformans/C. gattii*  *C. neoformans/C. gattii*  *C. neoformans* var. *grubii,* VNI  *C. neoformans* var. *grubii,* VNII  *C. gattii*, VGIV  *C. gattii*, VGI  *C. neoformans/C. gattii* | Gumbo et al. Pediatr Infect Dis J. 2002;21(1):54-6  Gumbo et al. Clin Infect Dis. 2001;32(8):1235-6  Boekhout et al. Microbiol. 2001;147(Pt 4):891-907  Chaka et al. J Infect Dis. 1997;176(6):1633-6  Gelfand. Centr Afr J Med. 1972;18:248-250  Matubu et al. Centr Afr J Med. 2015;61(1/4):5-11  Nyazika et al. J Med Microbiol. 2016;65(11):1281-1288  Boulware & Makadzange. N Engl J Med. 2017;376(11):1065-1071 |
| Botswana  (1817) | Gaborone  Botswana  Gaborone  Gaborone  Gaborone  Gaborone  Gaborone  Gaborone  Gaborone  Gaborone  Gaborone  Gaborone | 1018  4  3  73  29  193  105  25  9  22  27  1  1  19  2  38  5  17  25  9  1  16  175 | *C. neoformans/C. gattii*  *C. neoformans* var*. grubii,* A, VNI  *C. neoformans* var*. grubii,* A, VNB  *C. neoformans* species complex  *C. gattii* species complex  *C. neoformans/C. gattii*  *C. neoformans* var*. grubii,* A  *C. neoformans* var*. grubii,* A, VNB  *C. neoformans* var*. grubii,* A, VNI  *C. gattii,* C, VGIV  *C. neoformans/C. gattii*  *C. neoformans/C. gattii*  *C. neoformans/C. gattii*  *C. neoformans* var*. grubii,* VNI  *C. neoformans* var*. grubii,* VNII  *C. neoformans* var*. grubii,* VNB  *C. neoformans* v*. grubii,* VNI/VNB  *C. neoformans* var*. grubii,* VNI  *C. neoformans* var*. grubii,* VNBI  *C. neoformans* var*. grubii,* VNBII  *C. gattii,* VGI  *C. gattii,* VGIV  *C. neoformans/C. gattii* | Mullan et al. Pediatr Infect Dis. 2011;30(7):620-2  Litvintseva et al. PLOS ONE. 2011;6(5):e19688  Steele et al. Med Mycol. 2010;48(8):112-5  Bisson et al. S Afr Med J. 2008;98(9):724-5  Litvintseva et al. Genetics. 2006;172(4):2223-2238  Litvintseva et al. J Infect Dis. 2005;192(5):888-892  Bisson et al. Clin Infect Dis. 2013;56(8):1165-73  Rwegerera et al. Isr Med Assoc J. 2014;16:129-30  Navabi et al. J Int Assoc Provid AIDS Care. 2015;14(2):123-6  Chen et al. Mol Ecol. 2015;24(14):3559-3571  Fernandes et al. mBio. 2018;9(5):e02016-18  Mitchell et al. Pediatr Infect Dis. 2019;38:906-911 |
| South Africa  (20728) | Johannesburg  Durban  Sandringham  South Africa  Cape Town  Cape Town  Durban  Durban  Limpopo  Cape Town  South Africa  Johannesburg  Durban  Durban  Durban  Durban  Durban  Johannesburg  Tygerberg  Soweto  South Africa  Johannesburg  Pretoria  Johannesburg  Durban  Durban  Johannesburg  KwaZulu Natal  Cape Town  Bloemfontein  KwaZulu Natal  South Africa  Cape Town  South Africa  Cape Town  Kwa Zulu Natal  Kwa Zulu Natal  Soweto  Cape Town  KwaZulu Natal  KwaZulu Natal  South Africa  South Africa  Boksburg  Pretoria  Pretoria  South Africa  Pretoria  KwaZulu Natal  Durban  Johannesburg  Durban | 11861  314  9  487  67  8  6  1  1  1  4  2  31  2  7  514  36  1  26  1  1  1  5  1866  19  22  5  1  1  1  1  1  4  3  44  6  1  1  1  44  1  1  266  37  139  22  9  7  4  4  150  127  87  5  90  39  47  8  175  8  7  20  5  2  8  1  2  31  26  1  53  1  3817  15  102  13 | *C. neoformans* species complex  *C. gattii* species complex  *C. neoformans/C. gattii*  *C. neoformans/C. gattii*  *C. neoformans* var*. grubii,*A, VNI  *C. neoformans* var*. grubii,*A, VNB  *C. neoformans* var*. grubii,* A, VNII  *C. neoformans*, AD hybrids, VNIII  *C. neoformans/C. gattii*  *C. neoformans/C. gattii*  *C. neoformans/C. gattii*  *C. neoformans/C. gattii*  *C. neoformans* var*. grubii,* A, VNI  *C. neoformans* var*. grubii,* A, VNII  *C. neoformans* var*. grubii,* A, VNB  *C. neoformans/C. gattii*  *C. neoformans* var*. grubii,* A, VNI  *C. neoformans/C. gattii*  *C. neoformans/C. gattii*  *C. neoformans/C. gattii*  *C. neoformans/C. gattii*  *C. neoformans/C. gattii*  *C. neoformans/C. gattii*  *C. neoformans* var*. grubii,* A  *C. gattii,* B  *C. gattii,* C  *C. gattii* species complex  *C. neoformans/C. gattii*  *C. gattii,* C  *C. neoformans* var. *grubii*, A, VNI  *C. neoformans/C. gattii*  *C. neoformans/C. gattii*  *C. neoformans* var*. grubii,* A, VNI  *C. neoformans* var*. grubii,* A, VNII  *C. neoformans* var*. grubii,* A, VNI  *C. neoformans* var*. grubii,* A, VNII  *C. neoformans/C. gattii*  *C. neoformans/C. gattii*  *C. neoformans* species complex  *C. neoformans/C. gattii*  *C. neoformans/C. gattii*  *C. neoformans/C. gattii*  *C. neoformans/C. gattii*  *C. neoformans/C. gattii*  *C. neoformans* var. *grubii*, VNI  *C. neoformans* var. *grubii*, VNII  *C. neoformans* var. *grubii*, VNB  *C. neoformans* AD hybrids, VNIII  *C. gattii*, VGI  *C. gattii*, VGIV  *C. neoformans/C. gattii*  *C. neoformans/C. gattii*  *C. neoformans/C. gattii*  *C. neoformans/C. gattii*  *C. neoformans/C. gattii*  *C. neoformans/C. gattii*  *C. neoformans* var. *grubii*, VNII  *C. neoformans* var. *grubii*, VNI  *C. neoformans* var. *grubii*, VNII  *C. neoformans* var. *grubii*, VNB  *C. neoformans* var. *grubii*, VNI  *C. neoformans* var. *grubii*, VNII  *C. neoformans* var. *grubii*, VNB  *C. neoformans* var. *grubii*, VNI  *C. gattii*, VGI  *C. gattii*, VGIV  *C. neoformans/C. gattii*  *C. neoformans/C. gattii*  *C. neoformans/C. gattii*  *C. neoformans/C. gattii*  *C. neoformans/C. gattii*  *C. neoformans/C. gattii*  *C. neoformans/C. gattii*  *C. neoformans/C. gattii*  *C. neoformans/C. gattii*  *C. neoformans/C. gattii* | Meiring et al. AIDS. 2012;26(18):2307-14  Bhagwan & Naidoo. AIDS Res Treat. 2011;2011:180352  Govender et al. S Afr Med J. 2011;102(12)  Miglia et al. J Clin Microbiol. 2011;49(1):307-14  Jarvis et al. PLOS ONE 2010;8(7):e69288  Mitha et al. J Infect Dev Ctries. 2010;4(8)  Ramdial et al. Am J Dermatopathol. 2010;32(8):780-6  Ramdial et al. Int J Surg Pathol. 2011;19(3):386-92  Litvintseva et al. PLOS ONE. 2011;6(5):e19688  Jarvis et al. BMC Infect Dis. 2010;10:67  Botes et al. Microb Ecol. 2009;57(4):757-65  Swe Han et al. J Clin Pathol. 2008;61(10):1138-9  Ramdial et al. J Cutan Pathol. 2008;35(11)  Haddow et al. J Infect. 2008;57(1):82-4  Vawda e al. Br J Radiol. 2008;81(962):e53-6  Sing et al. Int J Gynecol Pathol. 2008;27(1):37-40  Sing & Ramdial. Am J Surg Pathol. 2007;31(10):1521-7  Morgan et al. Clin Infect Dis. 2006;43(8):1077-1080  Van Toorn et al. Eur J Paediatr Neurol. 2005;9(5):355-9  Karstaedt et al. Eur J Paediatr Neurol. 2005;9(5):355-9  Boekhout et al. Microbiol. 2001;147(Pt 4):891-907  Pantanowitz et al. J Infect. 2000;41(1):92-4  Botha & Wessels. J Clin Pathol. 1999;52(12):928-30  Meyer et al. Electrophoresis. 1999;20(8):1790-9  John et al. J Infect. 1998;36(2):231-2  Stead et al. J Infect. 1988;17(2):139-140  Sornum. BMJ Case Rep. 2012;2012:bcr2012007539  Jarvis et al. PLOS ONE. 2013;8(7):e69288  Moodley et al. Neuro-Ophthalmol. 2013;37(3):124-128  Ramkillawan et al. Int J Infect Dis. 2013;17(12):e1229-31  Jarvis et al. Clin Infect Dis. 2014;58(5):736-745  Boulware et al. N Engl J Med. 2014;370:2487-98  Van Wyk et al. J Clin Microbiol. 2014;52(6):1921-31  Jarvis et al. Clin Infect Dis. 2014;59(4):493-500  Adeyemi & Ross. J Int AIDS Soc. 2014;17(4 Suppl 3):19623  Loyse et al. J Infect. 2015;70(6):668-75  Govender et al. HIV Med. 2015;16(8):468-76  Jarvis et al. PLOS Pathog. 2015;11(4):e1004754  Drain et al. HIV Med. 2015;16(10)  Beale et al. PLOS Negl Trop Dis. 2015;9(6):e0003847  Rhodes et al. Genetics. 2017;207(1):327-346  Chen et al. mBio. 2017;8(2):e00166-17  Wake et al. Clin Infect Dis. 2018;66(5):686-692  Khan & Hiesgen. SA J Radiol. 2017;21(2):1215  Muranda et al. BMC Nephrology. 2018;19:94  Coetzee et al. PLOS ONE. 2018;13(6):e0198993  Kelly et al. World Neurosurg. 2018;118:5-8  Cassim et al. Afr J Lab Med. 2018;7(1)  Drain et al. Sci Rep. 2019;9:2687  Quan et al. PLOS ONE. 2019;14(12):e0225742  Wykowski et al. BMC Infect Dis. 2020;20:61 |
